# CD147 expression lacks prognostic relevance in esophageal cancer

**DOI:** 10.1007/s00432-022-03917-2

**Published:** 2022-01-08

**Authors:** Natalie Küsters, Katharina Grupp, Julia-Kristin Grass, Kai Bachmann, Tarik Ghadban, Faik G. Uzunoglu, Michael Tachezy, Daniel Perez, Matthias Reeh, Jakob R. Izbicki, Nathaniel Melling

**Affiliations:** General, Visceral and Thoracic Surgery Department and Clinic, University Medical Center Hamburg-Eppendorf, Hamburg, Germany

**Keywords:** CD147, Tissue microarray, Immunohistochemistry, Esophageal cancer, Esophageal adenocarcinoma, Esophageal squamous cell cancer

## Abstract

**Introduction:**

The role of CD147 as an important indicator of tumor prognosis remains controversially discussed in literature. We focused on the prognostic significance of CD147 expression in esophageal cancer patients. While some studies report that CD147 is an unfavorable prognostic factor in esophageal squamous cell carcinoma, others showed no significant correlation. However, only one study draws attention to the significance of CD147 in esophageal adenocarcinoma, which is one of the most rapidly increasing neoplasms in the western world.

**Methods:**

To finally clarify the impact of CD147 as a prognostic factor, especially for esophageal adenocarcinomas, we analyzed CD147 expression in a tissue microarray of 359 esophageal adenocarcinomas and 254 esophageal squamous cell cancer specimens. For the immuno-histochemical analysis, we used a primary antibody specific for CD147. Staining intensity and proportion of positive tumor cells were scored (negative, weak, moderate, strong staining). These findings were compared to normal esophageal tissue and correlated to the histopathological tumor phenotype and survival data.

**Results:**

CD147 expression was detectable in weak intensities in benign esophageal tissue (85.78%) and expressed in predominately moderate to strong intensities in esophageal cancer (88.34%). Strong CD147 immunostaining was linked to increased infiltration depth (*p* = 0.015) and differentiation (*p* = 0.016) in esophageal squamous cell cancer but revealed no significant correlation with histopathology of adenocarcinoma. Moreover, CD147 intensity was unrelated to overall survival in this collective for both subtypes of esophageal cancer.

**Conclusion:**

Thus, our data show that CD147 has no prognostic value, neither in esophageal adenocarcinoma nor squamous cell carcinoma.

## Introduction

The incidence of esophageal cancers has been increasing in recent years. One of the main causes of deaths is the fact, that clinical symptoms often only become apparent in late stages. There are marked geographical differences in the general incidence of esophageal carcinoma. In addition, esophageal cancer is one of the most aggressive types of carcinoma and it is the sixth leading cause of cancer death worldwide (Sung et al. 2021). A distinction is made between the two subtypes, esophageal squamous cell carcinoma (ESCC) and esophageal adenocarcinoma (EAC). In Western industrialized countries, the incidence of adenocarcinoma shows a significant and sustained rise over the last four decades, which makes esophageal cancer a major global health challenge (Smyth et al. 2017). Due to lack of early symptoms, patients are usually diagnosed in a locally advanced or metastasized stage, contributing to the fact that only about 40% of patients are suitable for surgery at the time of diagnosis. Thus, the 5-year overall survival rate is still poor (Sung et al. 2021). Identifying novel biological markers and tumorigenic pathways is vital for prediction of tumor behavior and may allow for personalized therapy.

The complex pathological process of tumor growth and spread includes several different pathways. Especially the interaction between tumor cells and host stromal cells is of particular interest. CD147, also known as extracellular matrix metalloproteinase inducer (EMMPRIN), is a widely expressed multifunctional glycoprotein (Biswas et al. 1995) and exerts essential roles in various cellular processes in several tissue types, such as the lung, thymus, retina, skin, cornea and nervous system (Iacono et al. 2007; Nabeshima et al. 2006). In various cancers, CD147 is highly expressed on the cell surface (Muramatsu and Miyauchi 2003; Yan et al. 2005; Yurchenko et al. 2006) and promotes the synthesis and secretion of matrix metalloproteinases in fibroblasts. This leads to degradation of the cancer cell matrix facilitating invasion and metastatic dissemination (Guo et al. 1997; Huang et al. 2015; Kanekura et al. 2002; Peng et al. 2016; Sameshima et al. 2000) on the one hand, and activation of tumor angiogenesis (Tang et al. 2004, 2005), and enhancement of cell survival signaling (Marieb et al. 2004) on the other. Several studies have described a correlation between increased CD147 expression and disease progression. Due to its membranous localization, CD147 has been suggested as a potential therapeutic target in several cancer types (Dean et al. 2009; Han et al. 2010; Hu et al. 2017; Zhang et al. 2018).

However, the prognostic role of CD147 in esophageal cancer remains controversial (Feng et al. 2013; Ishibashi et al. 2004; Wan and Wu 2012; Zhang et al. 2018; Zhu et al. 2011) with inconsistent results so far and nearly all available results reflecting patients with ESCC. The majority of studies suggest poor outcome for elevated CD147 expression (Huang et al. 2015; Wan and Wu 2012; Zhang et al. 2018; Zhu et al. 2011), whereas the largest cohort so far was not able to reveal any impact on recurrence-free survival (Ishibashi et al. 2004). Some studies linked high CD147 expression to advanced clinical stages (Huang et al. 2015; Wan and Wu 2012; Zhang et al. 2018) and lymph node metastasis (Feng et al. 2013; Huang et al. 2015; Wan and Wu 2012; Zhang et al. 2018; Zhu et al. 2011), while others reported no association with blood or lymphatic vessel invasion, tumor stage or distant metastasis (Cheng et al. 2006; Huang et al. 2015; Ishibashi et al. 2004). Some studies revealed an association between CD147 and invasion depth (Wan and Wu 2012; Zhu et al. 2011) and poor differentiation (Cheng et al. 2006; Feng et al. 2013; Huang et al. 2015; Wan and Wu 2012; Zhu et al. 2011), while others presented contradictory findings (Cheng et al. 2006; Ishibashi et al. 2004).

These controversies might be attributed to small and varying sample sizes and a limited level of evidence. To get more insights into the prognostic role of CD147 expression in esophageal cancer, especially in adenocarcinomas, we analyzed CD147 expression in a cohort of 359 adenocarcinomas and 254 squamous cell cancer specimens using a tissue microarray (TMA) with corresponding clinico-pathological data.

## Materials and methods

### Patients

Consecutive patients, which underwent radical esophagectomy at the Department of General, Visceral and Thoracic Surgery at the University Medical Center Hamburg-Eppendorf, were eligible for inclusion. Histopathological and clinical outcome data were collected and analyzed.

The tissue microarray technique was used to enable efficient analysis at the protein level. All specimens were analyzed according to a standard procedure, including complete embedding of the entire esophagus for histological analysis.

The study was approved by the Ethics Committee Hamburg and conducted in accordance with the Declaration of Helsinki. Usage of routinely archived formalin-fixed leftover patient tissue samples for research is approved by local laws and does not require written consent (HmbKHG, §12,1).

### Tissue microarray manufacturing

The TMA manufacturing process, as described by Mirlacher et al., was used for our study (Mirlacher 2010; Simon et al. 2004). We removed a cylinder measuring 0.6 mm from each patient’s primary, representative, formalin-fixed, and paraffin-embedded tumor block. The cylinders were then assembled in an empty array receiver block (Simon et al. 2004). The tissues were distributed among two TMA blocks. For internal controls, both TMA blocks also contained various control tissues, including normal esophageal tissue.

### Immunohistochemistry

For the immuno-histochemical analysis, all slides were de-paraffinized and exposed to heat-induced antigen retrieval for 5 min in an autoclave at 121 °C in pH 9 Tris–EDTA buffer. Next, a primary antibody specific for CD147 (mouse, monoclonal, Abcam #ab78106; at 1/150 dilution) was applied to the samples. All methods were carried out in accordance with the respective protocols. Visualization of the bound antibody was achieved with the EnVision Kit (Dako, Glostrup, Denmark). CD147 staining was found to be homogeneous in the analyzed tumor samples. Therefore, the tissue specimens were initially categorized semi-quantitatively into groups depending on CD147 expression.

The staining intensity (0, 1 + , 2 + , 3 +) and the proportion of positive tumor cells were analyzed separately for each tissue sample. These two parameters then served for final grading in negative, weak, moderate, and strong staining, as previously described by Juhnke et al. (2017). Negative scores indicated a complete absence of staining in the sample. A weak score was attributed when staining intensity was 1 + in up to 70% or 2 + in up to 30% of the tumor cells. Samples were classified as moderate with a staining intensity of 1 + in > 70%, 2 + in > 30% but in ≤ 70% or 3 + in ≤ 30% of the tumor cells. Strong scores showed a staining intensity of 2 + in > 70% or 3 + in > 30% of the tumor cells.

### Statistical analysis

Statistical analyses were performed using JMP 9 (SAS Institute Inc., NC, USA). To recognize associations between molecular parameters and tumor phenotype, contingency tables, and the *x*^2^-test were used. Kaplan–Meier curves were generated for survival analysis. Testing for survival differences between groups was performed with the Log-Rank test. A two-sided *p *value < 0.05 was considered statistically significant.

## Results

To elucidate the role of CD147, we analyzed cancer tissue from 359 esophageal adenocarcinoma patients and 254 esophageal squamous cell carcinoma patients.

### Technical implications

A total of 300 of 359 (83.6%) adenocarcinomas and 217 of 254 (85.8%) squamous cell carcinomas were interpretable for our TMA analysis. Reasons for non-informative cases included a complete lack of tissue or absence of unequivocal cancer tissue in the TMA section.

### Demographic and histopathological parameters

In the EAC group, 44 (14.7%) patients were female and 256 (85.3%) male. 67.0% were aged above 65 years. The majority of patients were detected in tumor stage pT3 (*n* = 180, 60.0%) and showed moderate (*n* = 114, 38.0%) to poor differentiation (*n* = 161, 53.7%).

In the ESCC group, tissue from 57 (26.3%) female and 160 (73.7%) male patients was available for analysis. 138 patients (63.5%) were older than 65 years and 123 (56.6%) were detected in tumor stage pT3. The largest share was moderately differentiated (*n* = 139, 64.0%). Median follow-up data for both groups were 17.3 (0–208; EAC) months and 12.2 (0–191; ESCC) months.

### CD147 immunostaining in esophageal cancers

**Fig. 1:**
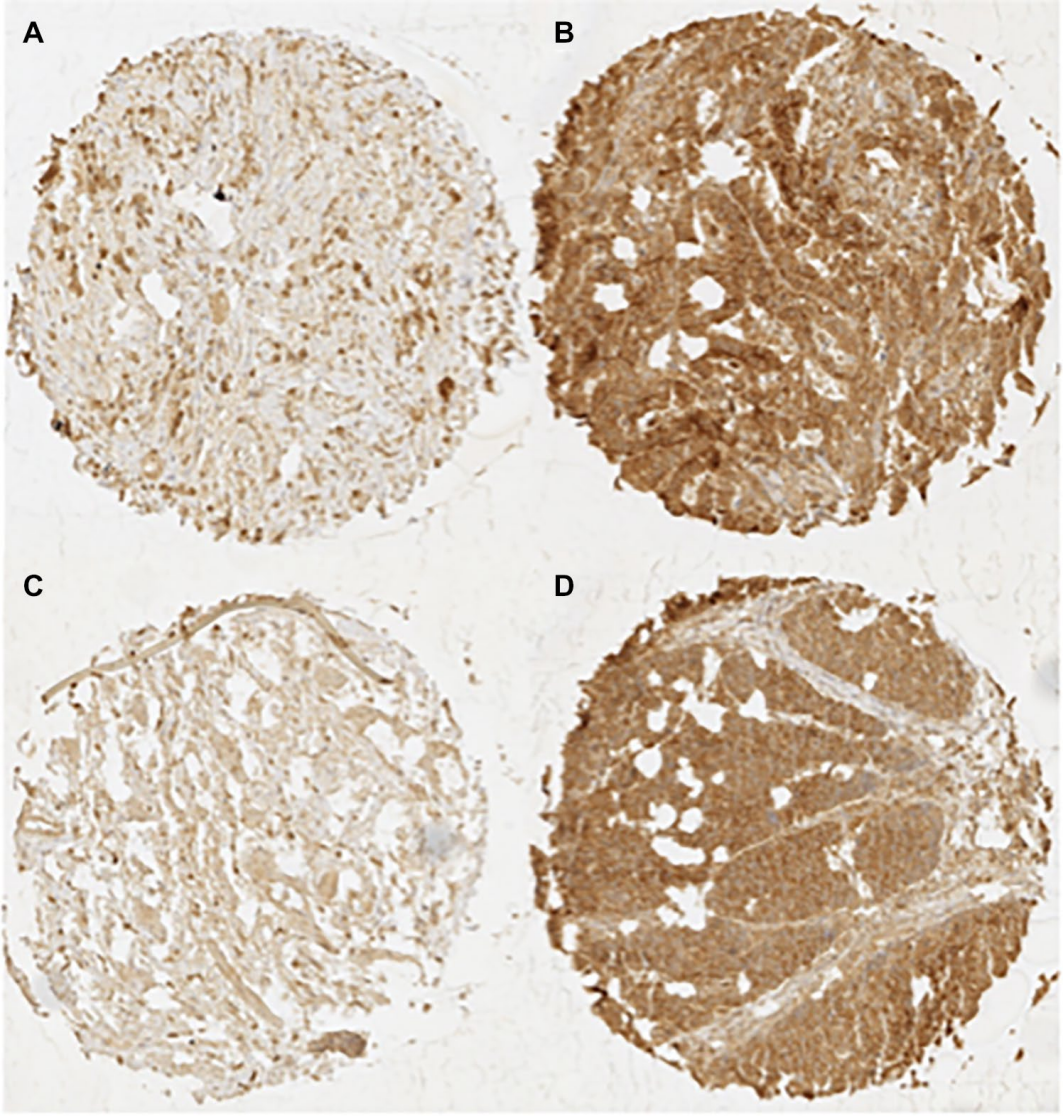
Immuno-histochemical images of CD147 staining. Images of weak and strong CD147 expression in EACs (**A** and **B**) and ESCCs (**C** and **D**)

CD147 immunostaining was typically absent or detectable in weak intensities in benign esophageal tissue and was predominantly localized in the cell membrane. In esophageal cancer, CD147 expression was higher than in benign cells. CD147 immunostaining was detectable in weak intensities in 11.7%, moderate intensities in 41.7%, and strong intensities in 46.7% of EACs, while CD147 immunostaining was weak in 14.2%, moderate in 50.5%, and strong in 35.3% of ESCC specimens. Figure 1 shows representative images of weak and strong CD147 immunostaining in EAC and ESCC.

### CD147 and histopathological tumor phenotype

For the subgroup of EAC, no significant correlation to infiltration depth (*p* = 0.513), lymphatic (*p* = 0.984) or distant dissemination (*p* = 0.643), tumor stage (*p* = 0.773) or differentiation (*p* = 0.135) was found (Table 1). Moreover, neither age nor gender had any impact on CD147 expression in these patients.

**Table 1:** Association of CD147 expression with clinico-pathological parameters in EAC

Immunostaining					
Parameter	Evaluable (*n*)	Weak (%)	Moderate (%)	Strong (%)	*P* value
Tumors	300	11.67	41.67	46.67	
Age (y)					
< 65	99	14.14	34.34	51.52	0.178
> 65	201	10.45	45.27	44.28	
Gender					
Male	256	11.33	40.63	48.05	0.509
Female	44	13.64	47.73	38.64	
Tumor stage					
pT1	63	9.52	34.92	55.56	0.513
pT2	35	11.43	34.29	54.29	
pT3	180	12.78	46.11	41.11	
pT4	20	10.00	40.00	50.00	
UICC stage					
I	62	8.06	37.10	54.84	0.773
II	38	13.16	44.74	42.11	
III	172	12.79	41.28	45.93	
IV	26	11.54	50.00	38.46	
Grading					
G1	15	6.67	46.67	46.67	0.135
G2	114	6.14	41.23	52.63	
G3	161	14.91	42.86	42.24	
G4	6	33.33	16.67	50.00	
R status					
R0	217	12.44	41.01	46.54	0.230
R1	75	9.33	46.67	44.00	
R2	3	0.00	0.00	100.00	
Nodal status					
pN0	90	11.11	40.00	48.89	0.984
pN1	52	11.54	46.15	42.31	
pN2	69	13.04	43.48	43.48	
pN3	87	11.49	40.23	48.28	
M stage					
M0	274	11.68	40.88	47.45	0.643
M1	26	11.54	50.00	38.46	

*EAC* esophageal adenocarcinoma, *UICC* Union internationale contre le cancer

*p* value indicates significance according to the *χ*^2^-test

In the ESCC cohort however, infiltration depth (*p* = 0.015) and differentiation (*p* = 0.016) were significantly associated with CD147 expression. In contrast, lymphatic (*p* = 0.889) and distant metastasis (*p* = 0.918), tumor stage (*p* = 0.275), gender and age showed no significant correlation with CD147 expression (Table 2). For both subgroups, no association between CD147 and resection margins was apparent.

**Table 2:** Association of CD147 expression with clinico-pathological parameters in ESCC

Immunostaining					
Parameter	Evaluable (*n*)	Weak (%)	Moderate (%)	Strong (%)	*p* value
Tumors	217	14.22	50.46	35.32	
Age (y)					
< 65	79	12.66	49.37	37.97	0.791
> 65	138	15.22	50.72	34.06	
Gender					
Male	160	13.75	50.00	36.25	0.892
Female	57	15.79	50.88	33.33	
Tumor stage					
pT1	40	25.00	40.00	35.00	**0.015**
pT2	42	21.43	42.86	35.71	
pT3	122	8.13	59.35	32.52	
pT4	13	15.38	23.08	61.54	
UICC stage					
I	53	24.53	41.51	33.96	0.275
II	58	12.07	46.55	41.38	
III	98	10.20	56.12	33.67	
IV	8	12.50	62.50	25.00	
Grading					
G1	3	0.00	33.33	66.67	**0.016**
G2	139	13.67	58.27	28.06	
G3	75	16.00	36.00	48.00	
G4	0	0.00	0.00	0.00	
R status					
R0	159	16.83	46.69	36.48	0.157
R1	51	16.00	48.00	36.00	
R2	7	14.29	85.71	0.00	
Nodal state					
pN0	98	16.49	46.39	37.11	0.889
pN1	51	9.80	54.90	35.29	
pN2	41	12.20	53.66	34.15	
pN3	27	18.52	48.15	33.33	
M stage					
M0	210	14.29	50.00	35.71	0.918
M1	7	14.29	57.14	28.57	

*ESCC* esophageal squamous cell cancer, *UICC* Union internationale contre le cancer

*p* value indicates significance according to the *χ*^2^-test. *p*-values in bold indicate statistical significance.

### CD147 and overall survival

**Fig. 2:**
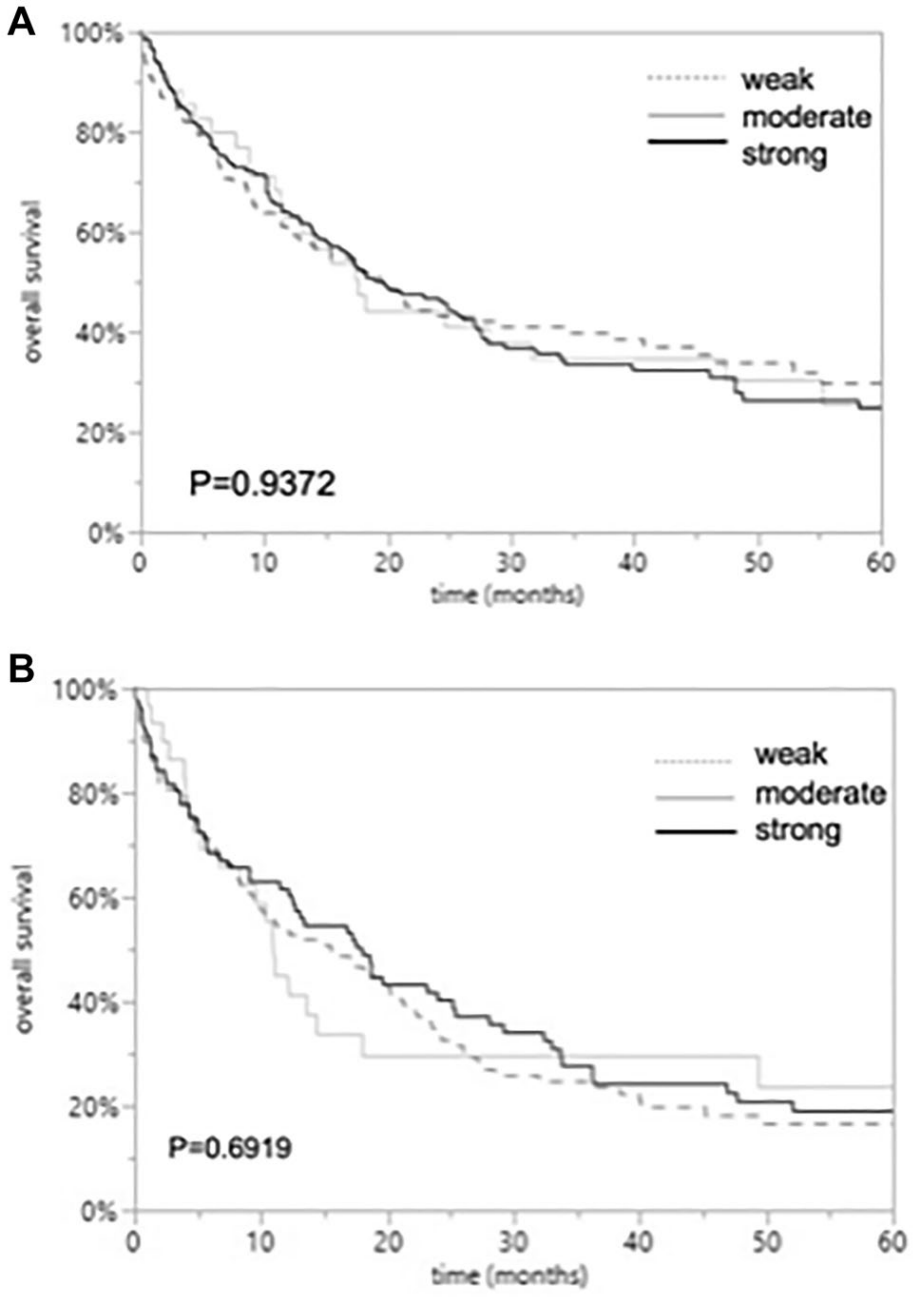
Overall survival in patients with esophageal cancers with weak, moderate, and strong CD147 immunostaining. p values indicate no significant difference between the groups according to Log-Rank test for EAC (*p* = 0.9372; **A**) and ESCCs (*p* = 0.6919; **B**)

Follow-up data were available for 196 EAC and 167 ESCC patients with informative CD147 data. CD147 immunostaining was not linked to overall survival of EAC and ESCC patients (*p* = 0.9372 and *p* = 0.6919). The relationship between CD147 immunostaining and clinical outcome of the patients is demonstrated in Fig. 2.

## Discussion

With these findings, we are able to clarify that CD147 has no prognostic relevance in esophageal adenocarcinoma or squamous cell cancer.

However, the comparative analysis of normal and neoplastic epithelium revealed an enhanced CD147 expression in most cancer specimens. While membranous CD147 was absent or weakly detectable in normal esophageal epithelium, strong staining was found in 46.7% EAC and 35.3% of ESCC patients. These results align with previous studies on CD147 expression in esophageal cancer, describing CD147 overexpression in malignant compared to non-malignant esophageal tissue (Cheng et al. 2006; Feng et al. 2013; Ishibashi et al. 2004; Wan and Wu 2012; Zhang et al. 2018; Zhu et al. 2011).

The upregulation of CD147 expression is supposedly associated with tumor progression in esophageal cancer (Landras et al. 2019). Numerous previous oncological studies dealt with CD147 due to its consistently high expression levels on the surface of various tumor types, such as malignant melanoma (Hu et al. 2017), colorectal (van der Jagt et al. 2006), breast (Marieb et al. 2004) and esophageal cancer (Cheng et al. 2006; Feng et al. 2013; Ishibashi et al. 2004; Wan and Wu 2012; Zhang et al. 2018; Zhu et al. 2011). CD147 has been shown to facilitate the secretion of extracellular matrix-degrading metalloproteases from fibroblasts, endometrial cells and cancer cells, such as MMP-1, MMP-3, MMP-9 and membrane type 1-MMP. In this way, carcinomatous cells can gain the ability to proliferate and migrate, promoting tumor growth and metastasis (Basset et al. 1990; Ellis et al. 1989; Xin et al. 2016).

Previously, the prognostic role of CD147 in esophageal cancer was controversially discussed in various studies (Feng et al. 2013; Ishibashi et al. 2004; Wan and Wu 2012; Zhang et al. 2018; Zhu et al. 2011). Almost all these studies reflected data from small numbers of esophageal squamous cell carcinoma patients. Since sample size is a significant factor in epidemiological studies, the limited cohort size in the above-mentioned studies might contribute to these controversies. In contrast, our cohort depicts the largest sample size so far, and our findings are in line with the largest currently published collective (Ishibashi et al. 2004), which did not find consideration in the meta-analysis.

In ESCC patients, we were able to find a significant correlation between CD147 expression, invasion depth and tumor differentiation, which is corroborated by previous studies (Cheng et al. 2006; Wan and Wu 2012; Zhu et al. 2011).

The only study dealing with esophageal adenocarcinoma, including 74 patients with type II/III adenocarcinoma of the esophagogastric junction (AEG) (Huang et al. 2015), also found a significantly higher rate of CD147 expression in EAC compared to non-malignant esophageal tissue. However, the authors proposed an association between high CD147 expression and advanced disease, for example, lymphatic and distant metastasis, which cannot be confirmed by our data. Our cohort did not reveal any links to any of the assessed clinico-pathological parameters. A lower total number of cases but a rather high fraction of advanced stages (46 of 74 patients staged UICC III/IV) in Huang’s cohort might explain the difference in these findings. In addition, these patients with advanced cancer did not undergo neoadjuvant treatment, which may well have had negatively influenced survival rates independent of CD147 staining (Huang et al. 2015).

However, due to its membranous localization and overexpression, CD147 may prove suitable as a therapeutic target. Previously, CD147 has been suggested as a promising target in several cancers, such as malignant melanoma (Hu et al. 2017) and esophageal cancers (Zhang et al. 2018). CD147 targeting has already been shown to suppress malignant melanoma in vitro and in vivo, highlighting the therapeutic potential of CD147 silencing (Hu et al., 2017). Additionally, overexpression of CD147 has been linked to the development of esophageal cancer and has been considered a potential target for anticancer therapies (Zhang et al. 2018).

The strength of this study is the cohort size for both ESCC and EAC, while published evidence is strongly limited and inconclusive, especially for esophageal adenocarcinoma. The monocentric character and a lost to follow-up ratio of 23.0% and 34.7% are its limitations.

In summary, CD147 expression was detectable in strong intensities in large fractions of our samples but lacked prognostic relevance. However, due to its overexpression, CD147 may represent a promising therapeutic target in a subset of esophageal cancer patients.

*p *value indicates significance according to the *χ*^2^-test. *EAC* esophageal adenocarcinoma, *UICC* Union internationale contre le cancer

*p *value indicates significance according to the *χ*^2^-test. *p *values in bold indicate statistical significance. *ESCC* esophageal squamous cell cancer, *UICC* union internationale contre le cancer

## Data Availability

The datasets analyzed during the current study are available from the corresponding author on reasonable request.
